# Glossopharyngeal schwannoma a rare case report: Diagnostic and surgical approach

**DOI:** 10.1016/j.ijscr.2023.108629

**Published:** 2023-08-06

**Authors:** Essa Tawfeeq, Lulwah AlSaidan, Jafar Hayat, Bodour AlAbdulrahim, Mariam Sarkhouh, Mutlaq Al-Sihan

**Affiliations:** Department of Otolaryngology, Head and Neck Surgery, Zain Hospital, Shuwaikh Medical Area, Kuwait

**Keywords:** Schwannoma, Glossopharyngeal nerve, Neck mass, Rare

## Abstract

**Introduction and importance:**

Schwannomas are tumors of the nerve sheath that consist of Schwann cells that are often described as slow-growing. Glossopharyngeal schwannomas are rare tumors present in the region of the posterior fossa, with limited case reports present in literature. While patients may present asymptomatically, some present with vestibulocochlear symptoms or lower cranial nerve dysfunction.

**Clinical presentation:**

We report an extremely rare case of a left para-pharyngeal carotid space glossopharyngeal schwannoma in a 26-year-old female. The presentation was a 3-month left sided neck swelling and a hoarse voice. Radiological investigations were completed (neck ultrasound; CT; MRI scans). Investigations revealed a solid lesion measuring about 29 × 10 mm. The final decision was to excise the mass under microsurgery.

**Clinical discussion:**

CN 9–11 schwannomas are often called jugular foramen schwannomas. Intraoperatively, these get differentiated as glossopharyngeal schwannomas. Diagnosis involves a physical examination, a detailed history, audiological assessments, and radiological investigations. While MRI scans are known as the most effective pre-operative diagnostic test, cases are in majority discovered intra-operatively. Surgical excision is the recommended approach. Post-operative recurrence is rare. Pre-operative diagnosis is often difficult due to the rarity and similarly presenting differential diagnoses.

**Conclusion:**

Schwannomas of the glossopharyngeal nerve are extremely rare tumors that may present with lower cranial nerve or vestibulocochlear deficits. Magnetic resonance imaging is a useful tool in diagnosing this unordinary tumor. This case report intends to provide further data regarding the clinical presentation, the patient population, and the diagnostic and surgical approach in dealing with this incredibly rare tumor.

## Highlights

• Schwannomas are encapsulated, benign tumors of the nerve sheath originating from Schwann cells.
• The symptoms of glossopharyngeal schwannoma are non-specific, until the tumor gets a fairly large size.
• The diagnosis of glossopharyngeal schwannoma is confirmed intraoperatively.

## 1 Introduction

A schwannoma, also known as a neurinoma, is a type of a nerve sheath tumor that is formed by Schwann cells. These cells form an insulating layer around the peripheral nerves, representing approximately 7 % to 10 % of all primary intracranial tumors [1]. Below 50 cases of glossopharyngeal schwannomas have ever been reported [2].

Schwannomas can grow on both peripheral nerves and nerve roots; with a notable example being vestibular schwannomas and jugular foramen schwannomas. These originate from the ninth, tenth, and eleventh cranial nerves [3,4]. Among the posterior fossa tumors, the extremely rare glossopharyngeal schwannomas often present with an increased likelihood towards males and usually occur during the third to fifth decades of life. Symptoms of patients with schwannomas may not manifest until the tumor attains a fairly large size, and these symptoms may vary from subtle to severe [1,4]. The diagnosis of glossopharyngeal schwannomas is usually made once the tumor's attachment to the ninth nerve is seen at surgery [1]. It is necessary for a patient to consult with an ENT surgeon who is familiar with the risks and benefits of the treatment options [1].

The objective of this case report intends to provide further literature on this incredibly rare tumor and to demonstrate yet another example of how a patient may present, alongside demonstrating the steps taken to adequately manage the case. This case report brings up one of the only reported glossopharyngeal schwannomas found in the Middle East. This work has been reported in line with the SCARE 2020 criteria [5].

## 2 Presentation of case

A 26-year-old female presented in June 2021 with a three-month history of a left sided neck mass. The patient described her presentation as a fullness in her throat. Later, she noticed a painful left sided neck mass associated with hoarseness of voice. This mass had grown to approximately 2 cm in size when she had first sought to seek medical attention. The patient denied any history of snoring, dysphagia, odenophagia, fever, or weight loss. There were no neurological or otological symptoms.

The patient was previously healthy with no past medical history. She underwent previous surgeries including a sleeve gastrectomy, abdominoplasty, and a laparoscopic cholecystectomy. She is non-married and has been a smoker for 10 pack years. She has no history of any drug allergies and has no family history of the same complaint of any genetic or immunological disorders. It is to be noted that patient comes from a middle-class background.

On examination, the patient was healthy looking, conscious, alert and oriented to time, place and person. Her vitals were unremarkable and she was afebrile. Examination of the nose, throat, paranasal sinuses, and tympanic membranes were normal. Tuning fork tests (Rinne and weber tests) were normal. No spontaneous nystagmus was noted. Her neurological examination was normal and her cranial nerves were intact. Examination of her neck showed a tender left sided neck mass, anterior to the sternocleidomastoid muscle, of 2 × 2 cm in size, firm, with normal skin above it. There were no enlarged cervical lymph nodes. Her full blood count, urea and electrolytes, fasting blood sugar, cholesterol, and triglycerides, were all within normal limits.

Ultrasound of the neck was done that showed a well-defined oval shaped lesion seen just posterior to the left external carotid artery of deeply hypoechoic texture, showing no significant internal nor peripheral vascularity, measuring about 29 × 10 mm. CT scan with contrast of the neck showed left carotid space mass. MRI neck was done that showed a left para-pharyngeal carotid space solid lesion most likely either a carotid body tumor or a schwannoma for further workup. FNAC was not advised as it is contraindicated in cases of carotid body tumor. A 24-hour urinary metanephrines test was normal, which rules out paraganglioma. A decision to proceed for surgical excision was made.

A final decision was made to proceed with a surgical excision of the tumor once the patient's informed consent was acquired. The patient was briefed about the surgical protocol and the risks such as bleeding, damage to nearby structures, or infection. The patient was booked for surgery at December 2021 for an elective excision of the mass.

A left hockey stick incision was made 2 cm below the mandible, extended from the midline of the neck to 1 cm behind the sternocleidomastoid muscle. Incision of the subcutaneous tissue, superficial, deep and investing layer, platysma was done. Flaps were raised superiorly and inferiorly. Identification and incision of the carotid sheath layer was done. The carotid artery and internal jugular vein were mobilized and separated from the mass. The vagus nerve was identified, which was away from the mass. Ultimately, glossopharyngeal schwannoma capsule identified and excision of entire mass was done with the use of microscope. The histopathology result came out as glossopharyngeal schwannoma.

The patient was routinely followed up after being discharged with standard pain management, and was seen 2 weeks, 3 months, 6 months, and 1 year post-op, and had no complications or complaints. The wound site also healed adequately.

**Fig. 1:**
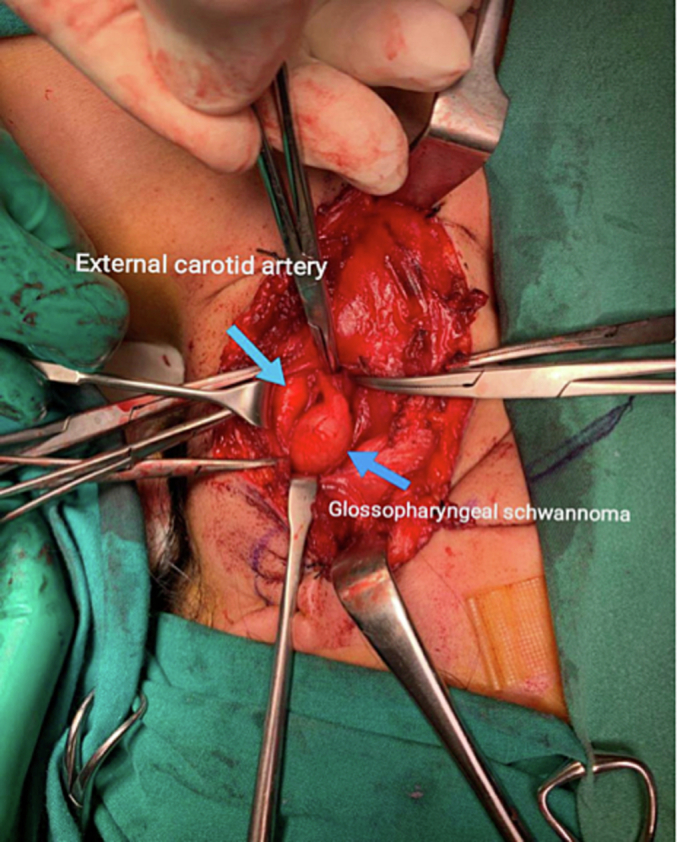
Shows the glossopharyngeal schwannoma and the external carotid artery.

**Fig. 2:**
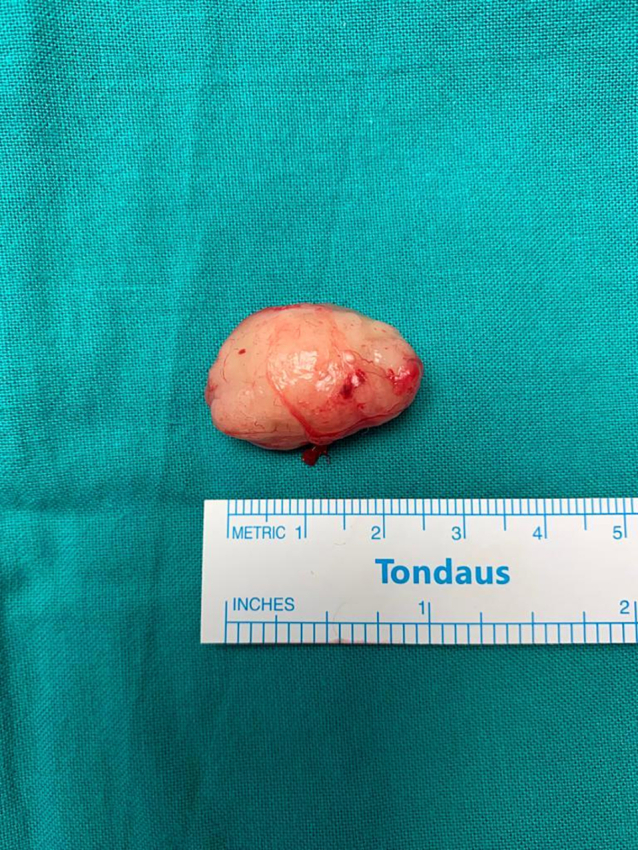
Shows the glossopharyngeal schwannoma after surgical excision.

**Fig. 3:**
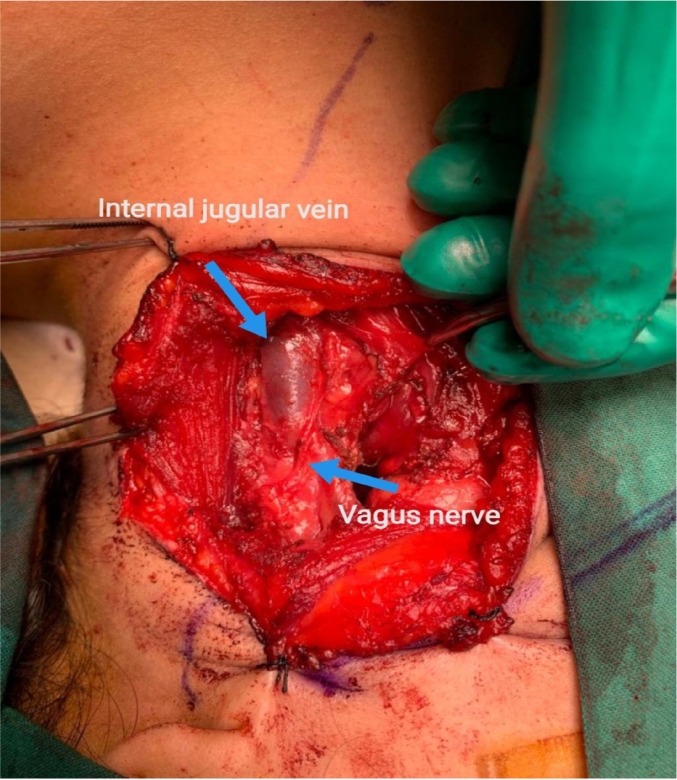
Shows the internal jugular vein, vagus nerve after glossopharyngeal schwannoma excision.

**Fig. 4:**
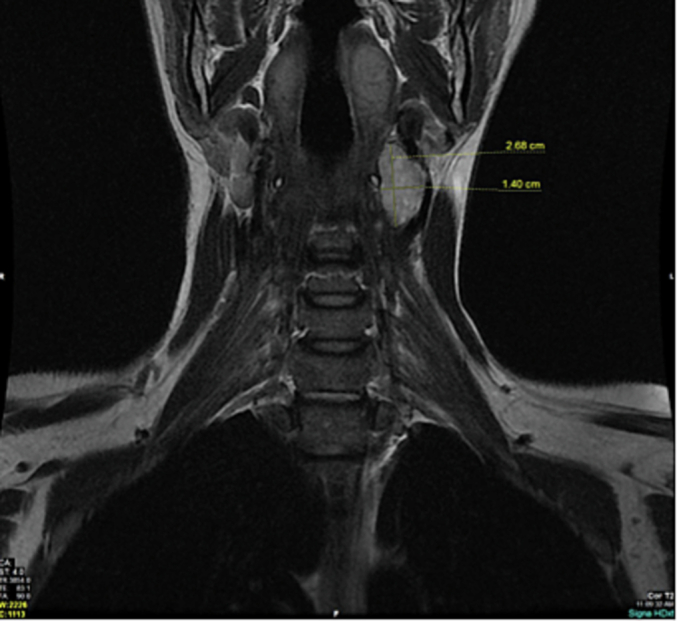
Coronal image of MRI showing well defined oval lesion at the carotid bed, showing intermediate to high signal on T2 fat spin echo sequence. Abutting the surrounding vasculature with mild mass effect. Measuring (1.4 × 1.4 × 2.68 cm –on AP, ML, CC dimensions).

All activities and consultations mentioned have been conducted in Kuwait without any outsourcing of resources (Fig. 1, Fig. 2, Fig. 3, Fig. 4).

## 3 Discussion

The glossopharyngeal nerve emerges from the cranial part of the medulla and leaves as 3 or 4 roots in the groove between the olive and the inferior cerebellar peduncle directly superior to the vagus nerve. It unites into a single nerve bundle that enters the jugular foramen through its neural compartment [2]. Most glossopharyngeal schwannomas described in the literature emerge from the intracranial element, commonly in the lower cerebellopontine angle. In addition, most of the very limited reported extracranial schwannomas were lesions that originated intracranially and advanced secondarily as extracranial extensions [6]. Usually, schwannomas of the 9th–11th cranial nerves are called jugular foramen schwannomas without specification. Only intraoperatively these are determined to be glossopharyngeal schwannomas.

Glossopharyngeal schwannomas are considered extremely rare, especially in the absence of underlying neurofibromatosis [6,7]. In literature, it is more common in men compared to women by approximately 1.5:1 and is predominantly on the right side. Patients can present with hearing loss, loss of taste, hoarseness, pain, and compressive symptoms. However, it is usually asymptomatic due to the contralateral nerve compensation [6,7]. Symptoms may not appear until the lesion has reached a reasonably large size, and vary from mild to severe. Schwannomas may occur spontaneously, or in the context of a familial tumor syndrome such as neurofibromatosis type 2 (NF2), schwannomatosis and Carney's complex. They very rarely undergo malignant transformation. Central to the pathogenesis of these tumors is loss of the function of Merlin, a cytoskeletal tumor suppressor protein [8].

**Fig. 5:**
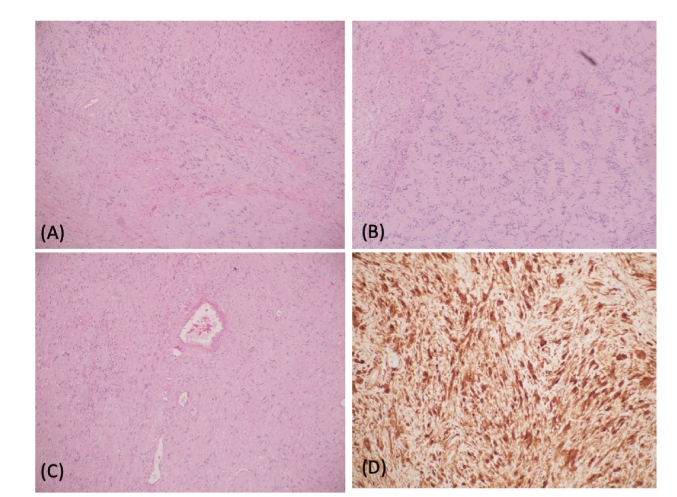
Pathological figure of the sample, showing the following: Schwannoma (A) Biphasic spindle cell lesion formed by: hypercellular “Antoni A” areas and less cellular “Antoni B” areas. (B) Nuclear palisading around fibrillary process (Verocay bodies). (C) Thickened hyalinized blood vessels walls are seen. (D) Strong S100+ immunoreactivity seen in the majority of the tumor cells.

The diagnosis of Glossopharyngeal Nerve Schwannoma involves physical examination, medical and familial screening for conditions such as NF2, and audiometric and hearing tests such as: otoscopy, Rinne and Weber tests, tympanometry, acoustic reflex, static acoustic measures, and auditory brainstem response and otoacoustic emissions. Relevant radiological investigations include: Computerized tomography (CT) scans of the head and neck with contrast, magnetic resonance imaging (MRI) with gadolinium, MR angiography, and also possibly positron emission tomography (PET) scans to rule out malignancy there is a metastases. Tissue biopsy is considered to be the gold standard in diagnosing glossopharyngeal schwannoma [9]. The pathological figure of the tissue biopsy can be seen in Fig. 5 below.

Since specific clinical symptoms and radiological findings can be absent in most cases of glossopharyngeal schwannomas as presented here, the diagnosis might be challenging [10]. The differential diagnosis of glossopharyngeal schwannomas includes vestibular schwannomas, vagus nerve schwannoma, neurogenic tumors, paraganglioma from the carotid body, neurofibromas, aneurysms, pseudoaneurysms of the carotid artery and branchial cleft cysts [6].

Surgical excision is recommended for treating glossopharyngeal nerve schwannoma with preservation of CN9. In our case, surgical excision with a left hockey stick incision in the neck was done below the mandible. The glossopharyngeal nerve was identified intraoperatively. Most common complication of surgery is permanent nerve damage, so identification of the nerve must be done to avoid such complication.

The prognosis of glossopharyngeal nerve schwannoma is generally positive, especially with preservation of the underlying nerve. In such cases, recovery from cranial nerve dysfunction after surgery is also fairly acceptable, although bilateral tumors and the positive history of neurofibromatosis can affect the outcome adversely. However, an early diagnosis and prompt management of the tumor will help improve the outcome. The chance of recurrence of glossopharyngeal nerve schwannoma after surgical excision is rare [11].

## 4 Conclusion

This report intends to provide another piece of literature that covers the incredibly rare condition of glossopharyngeal schwannomas, providing future practitioners insight towards the presentation of patients and differential diagnoses to note. This case report also provides insight towards the difficulty of diagnosing this condition, and discusses the ways it is diagnosed, with magnetic resonance imaging being the best tool pre-operatively and a tissue biopsy intra-operatively. As a conclusion, this case reports hopes to further the knowledge of this condition for the presentation, patient population, diagnostic, and surgical approach for this incredibly rare tumor by providing a peri-operative case report of a patient with the condition.

## Ethical approval

Ethical approval was waived by the author's institution.

## Funding

This research did not receive any specific grant from funding agencies in the public, commercial, or not-for-profit sectors.

## Author contribution

Dr. Essa Tawfeeq; data analysis and contribution

Dr Lulwah AlSaidan; data collection, data analysis, and write up.

Dr Jafar Hayat; data collection and write up.

Dr Bodour AlAbdulrahim; data collection and write up.

Dr Mariam Sarkhouh; data collection and write up.

Dr Mutlaq Al-Sihan; data analysis and contribution.

## Guarantor

Dr Lulwah AlSaidan

## Research registration number

N/A

## Consent

Written informed consent was obtained from the patient for publication of this case report and accompanying images. A copy of the written consent is available for review by the Editor-in-Chief of this journal on request.

## Conflict of interest statement

There is no conflict of interest to declare by any of the authors of this study.
